# Case Report: A case of Lynch syndrome-related glioblastoma with coexisting *MSH2* splicing defect and *MSH6* frameshift mutation

**DOI:** 10.3389/fonc.2026.1727445

**Published:** 2026-01-30

**Authors:** Liwei Huang, Xiaochun Tang, Demin Cao, Yulei Li, Xiaoying Zhu

**Affiliations:** 1 Youjiang Medical University for Nationalities, Baise, Guangxi, China; 2 Baise People’s Hospital, Baise, Guangxi, China; 3 Key Laboratory of Molecular Pathology in Tumors of Guangxi, Baise, Guangxi, China; 4 Clinical Pathological Diagnosis & Research Center, The Affiliated Hospital of Youjiang Medical University for Nationalities, Baise, Guangxi, China

**Keywords:** dMMR, gene variants, glioblastoma, Lynch syndrome, MSI-H

## Abstract

This case report describes a 38-year-old Chinese male with Lynch syndrome (LS)-associated glioblastoma (GBM), harboring concurrent germline NM_000251.3:c.942 + 3A>T and somatic NM_000179.3:c.3261dup mutations. The patient presented with progressive headaches, and imaging revealed a right frontal lobe mass with features suggestive of high-grade glioma. Histopathological and molecular analyses confirmed glioblastoma (WHO grade IV), microsatellite instability-high (MSI-H), and mismatch repair deficiency (dMMR). Familial cancer history, including colorectal and gallbladder malignancies in first-degree relatives, aligned with LS diagnostic criteria. The co-occurrence of *MSH2* splicing disruption and *MSH6* frameshift mutation synergistically exacerbated genomic instability, highlighting a potential mechanism for LS-driven gliomagenesis. This case underscores the importance of genetic screening in young-onset or familial GBM patients, advocates for integrating molecular profiling into therapeutic decision-making, and expands the understanding of LS-associated CNS tumorigenesis.

## 1 Introduction

Glioblastoma (GBM), classified as adult diffuse glioma by the World Health Organization (WHO) in 2021, represents the most aggressive and prevalent primary malignant brain tumor, predominantly affecting males with an incidence of 14.2% (1). GBM is characterized by rapid growth, strong aggressiveness, and poor prognosis. Despite aggressive multimodal therapy, the median survival of patients is only 12–15 months (2, 3).

Lynch syndrome (LS), an autosomal dominant hereditary cancer predisposition syndrome, is primarily attributed to defects in DNA mismatch repair (dMMR), conferring elevated risks of colorectal and endometrial carcinomas (4). Nevertheless, LS is associated with an increased lifetime risk of extraintestinal malignancies, including tumors of the ovaries, stomach, small intestine, hepatobiliary system, urinary tract, and central nervous system (CNS) (5). The molecular basis of LS involves germline mutations of DNA mismatch repair genes, primarily in *MLH1*, *MSH2*, *MSH6*, and *PMS2* (6). Among them, *MSH2* and *MSH6* form a heterodimer complex (MutSα), which plays a crucial role in recognizing base mismatches and small insertion-delete rings during DNA replication (7). Mutations in these genes result in defective MMR and lead to microsatellite instability (MSI), a condition that contributes to carcinogenesis. Consequently, MSI is frequently utilized as one of the biomarkers for diagnosing LS (8).

GBM is characterized by an immunosuppressive microenvironment, rendering standard immunotherapy largely ineffective. A critical gap in current research is the identification of rare molecular subgroups that may defy this paradigm. While the association between Lynch syndrome and brain tumors (particularly GBM) remains understudied, accumulating evidence highlights a significant risk elevation. A prospective cohort study of 288 LS families in Denmark reported that 14% of families developed primary brain tumors, with glioblastoma constituting 56% of cases. Notably, *MSH2* mutation carriers exhibited a 2.5% cumulative lifetime risk for brain tumors, a risk substantially higher than that of *MLH1* or *MSH6* carriers (9). Recent analyses further indicate that LS patients face a 2- to 4-fold increased incidence of gliomas compared to the general population, underscoring the need for targeted surveillance in high-risk cohorts and the establishment of routine screening guidelines (9, 10). The following case exemplifies how integrated molecular profiling combined with a detailed family cancer history can unveil LS-associated glioblastoma, providing mechanistic insights into MMR deficiency-driven tumorigenesis.

## 2 Case presentation

**Figure 1:**
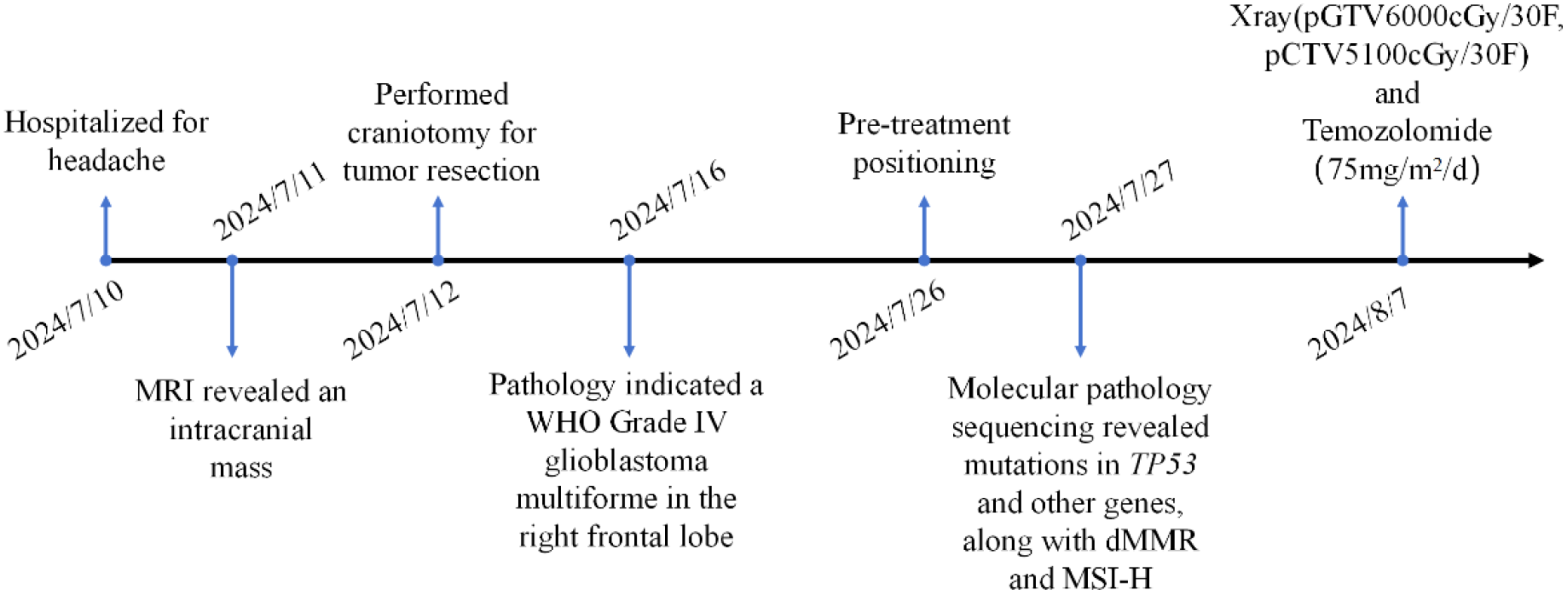
Timeline of diagnosis and treatment for this patient.

**Figure 2:**
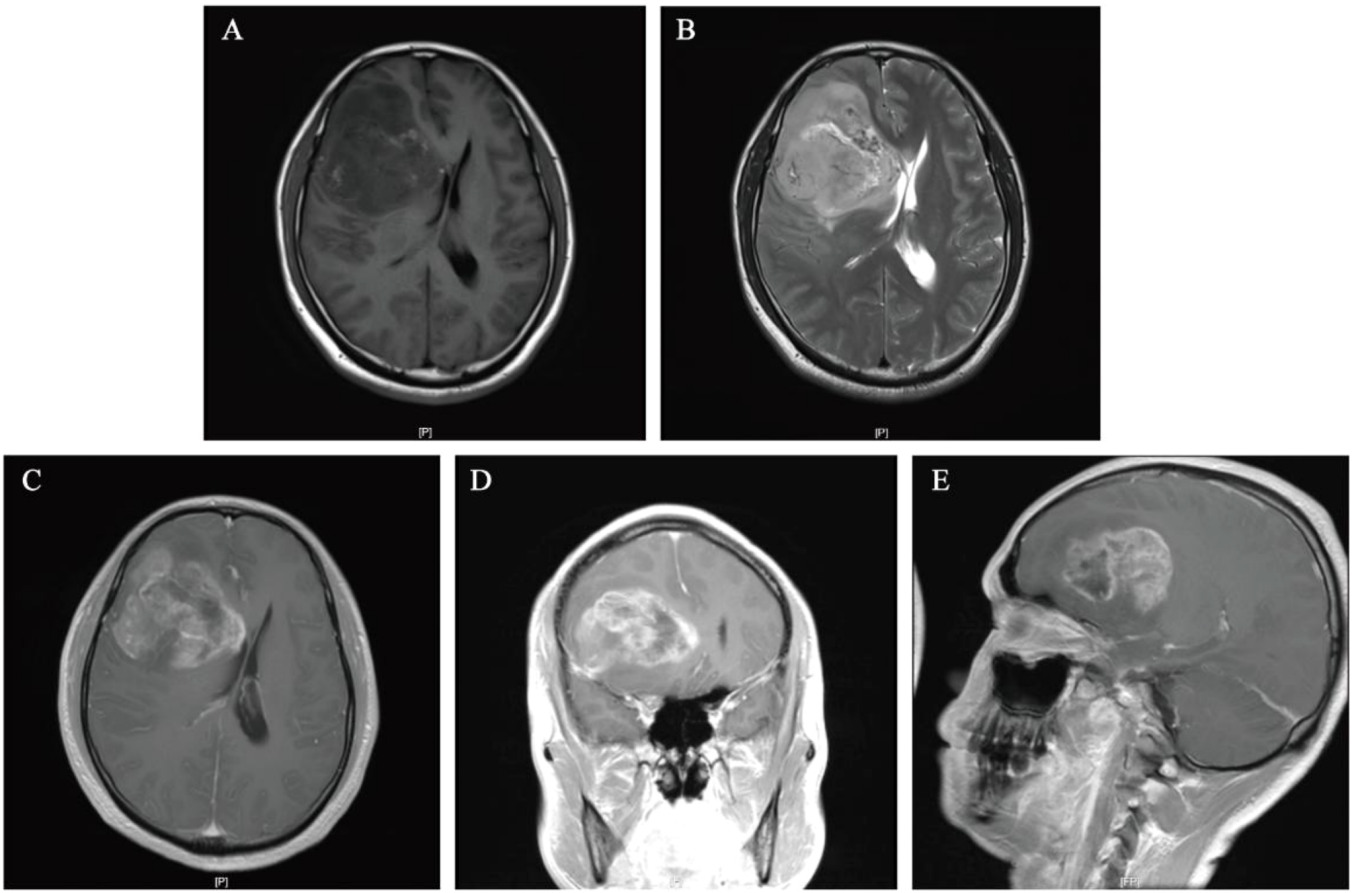
Preoperative MRI images of head. **(A, B)** T1-weighted sequences and T2-weighted sequences. **(C-E)** The lesion exhibits pronounced enhancement at its margins and within its solid components on T1-weighted contrast-enhanced axial, coronal, and parasagittal MRI.

A 38-year-old male presented to Baise People’s Hospital on July 10, 2024, with a 10-day history of progressive right frontal headache (A brief timeline is shown in **Figure 1**). The patient reported a 10-day history of right frontal headache characterized by paroxysmal throbbing pain with variable duration. The headache was accompanied by concurrent respiratory symptoms including coughing and sputum production. Temporary symptomatic relief was achieved through oral ibuprofen administration. Subsequently, the patient sought medical attention at a local hospital, though specific treatment details remain undocumented. Following this intervention, the respiratory symptoms (cough and sputum production) showed improvement, however, the headache persisted without complete resolution. The patient manifested acute-onset nausea and vomiting upon hospital admission. Clinical assessment revealed an unremarkable physical examination with preserved consciousness (Glasgow Coma Scale 15), though self-reported sleep quality indicated inadequate nocturnal rest. Notably, no neurological deficits or systemic abnormalities were detected during comprehensive evaluation. Neuroimaging evaluation utilizing a 1.5 Tesla MRI system with multiparametric sequences (contrast-enhanced T1-weighted imaging, diffusion-weighted imaging [DWI], magnetic resonance spectroscopy [MRS], dynamic susceptibility contrast [DSC], and diffusion tensor imaging [DTI]) demonstrated a complex right frontal lobe mass measuring 68×71×51 mm. The lesion exhibited heterogeneous cystic-solid architecture with the following characteristics: (1) T1-weighted imaging: Hypointense solid components with scattered hyperintense foci suggesting hemorrhagic conversion. (2) T2-weighted imaging: Predominantly hyperintense signal with peripheral perilesional edema. (3) Contrast enhancement: Irregular garland-like enhancement pattern with inflow-type time-intensity curve on DSC perfusion. (4) Structural invasion: Extension to right insular cortex and hippocampal formation with effacement of adjacent sulcal anatomy. (5) Mass effect: 11mm midline shift with ipsilateral ventricular compression (**Figures 2A–E**). Following the radiographic diagnosis of a malignant intracranial neoplasm, the patient underwent craniotomy for tumor resection on July 12, 2024. Surgical specimens underwent immediate fixation in 10% neutral buffered formalin followed by standardized processing for light microscopic evaluation.

**Figure 3:**
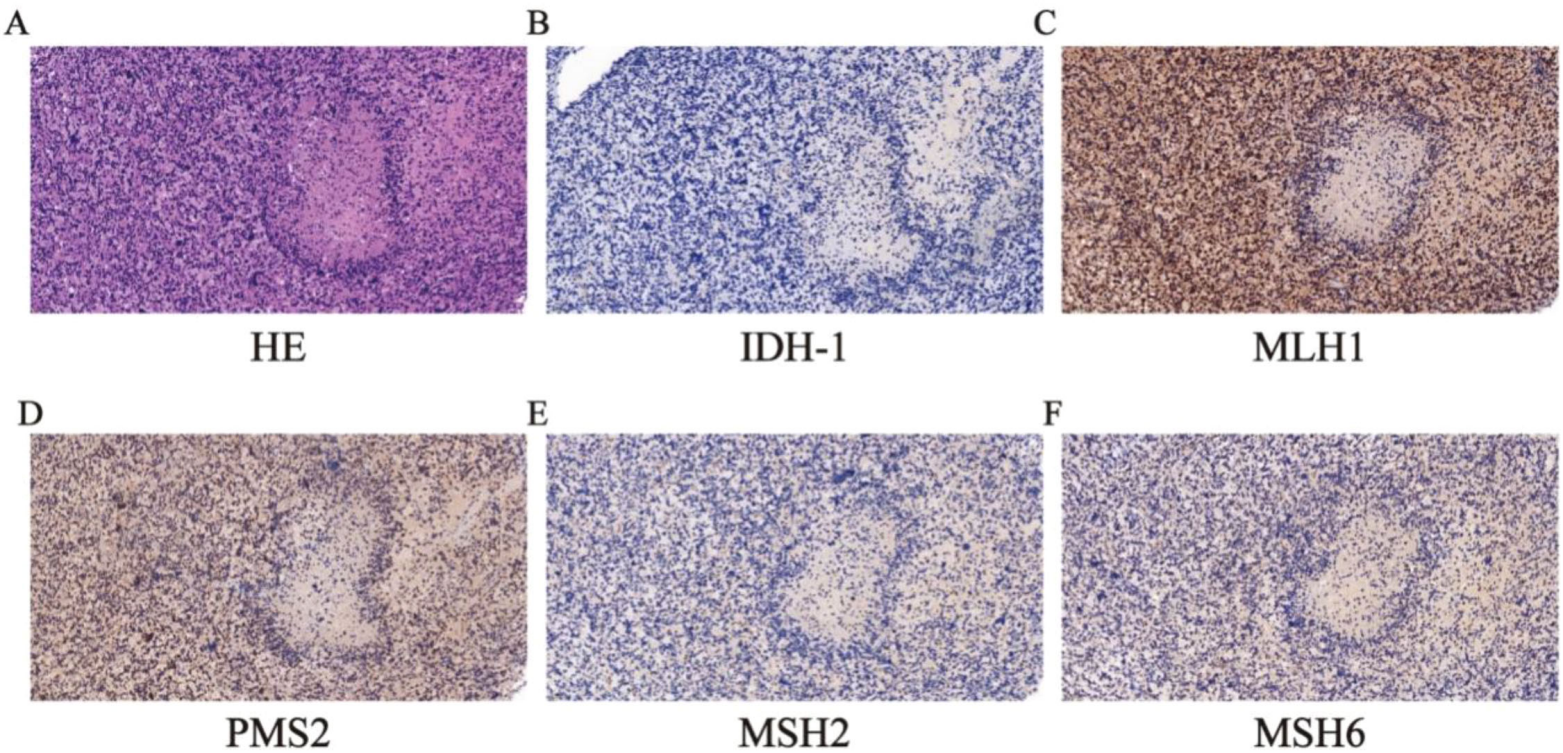
Pathological pictures of glioblastoma. **(A)** H&E staining showed the tumor. **(B)** IDH protein was wild type (20×). **(C, D)** The immunohistochemistry of MLH1 and PMS2 were positive in the tumor (20×). **(E, F)** The immunohistochemistry of MSH2 and MSH6 were absent in the tumor (20×).

**Figure 4:**
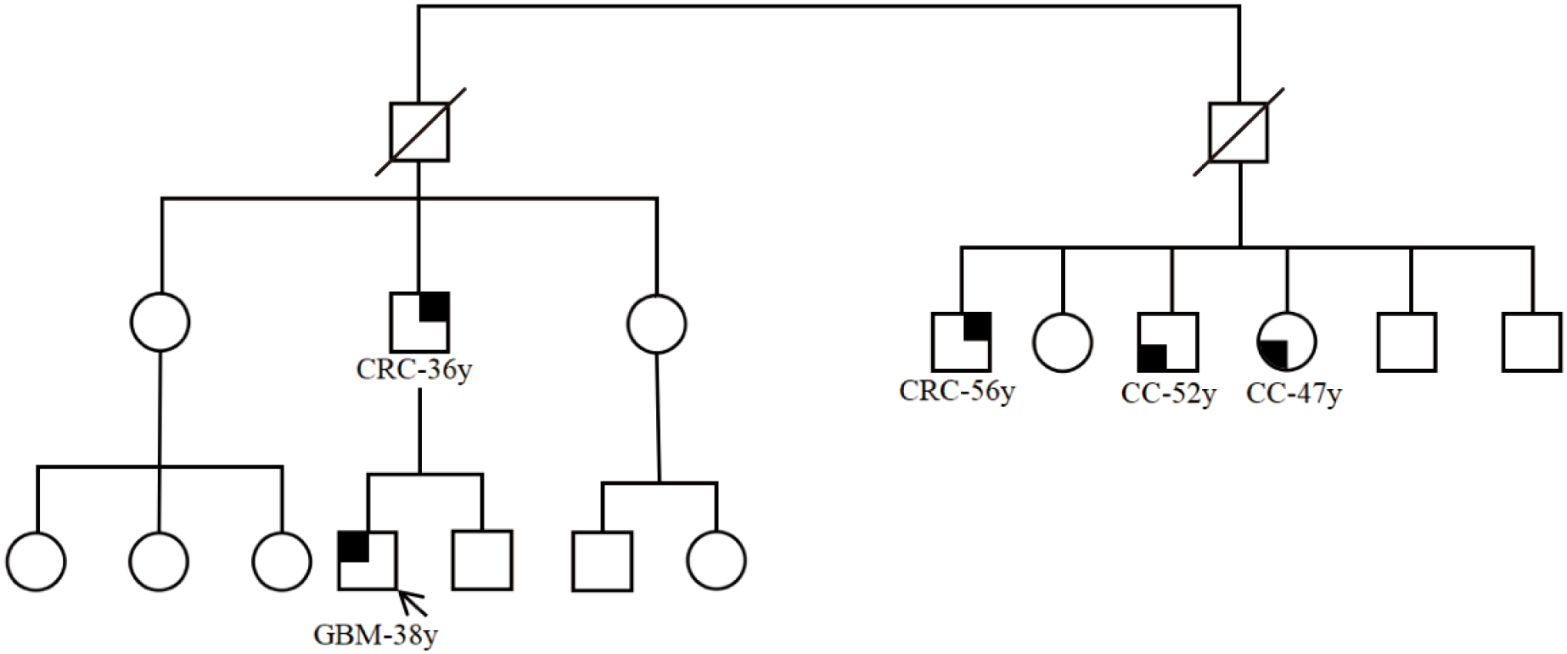
Genealogy of the family. Circles indicate represented to females, squares indicate males, the diagnosis and current age are below the symbols. CRC, colorectal cancer (symbols with filled right upper quadrant); GBM, glioblastoma (symbols with filled left upper quadrant); CC, colon cancer (symbols with blanked left lower quadrant).

**Figure 5:**
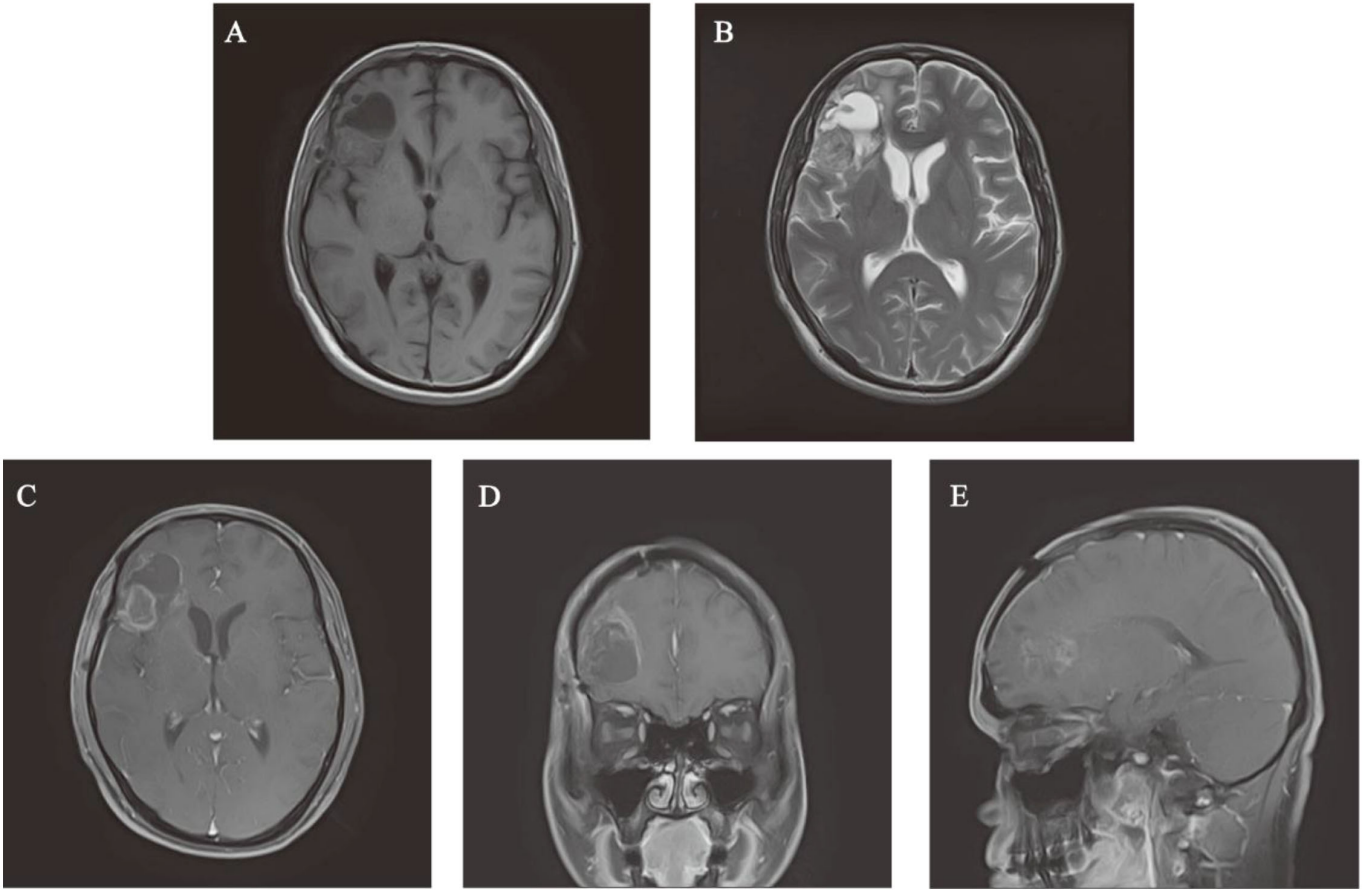
Postoperative MRI images of head. **(A, B)** T1-weighted sequences and T2-weighted sequences. **(C–E)** The surgical area lacks notable enhancement on post-contrast T1-weighted MRI, shown in axial, coronal, and parasagittal views.

Histopathological examination revealed hypercellular tumor tissue marked by nuclear pleomorphism, multinucleated giant cells, frequent mitotic figures (including atypical forms), and geographic necrosis (**Figure 3A**). The immunohistochemistry showed oligo-2, Glial Fibrillary Acidic Protein, P53 and ki-67 were positive and IDH was wild-type (**Figure 3B**). Histopathological diagnosis confirmed IDH-wildtype glioblastoma (CNS WHO Grade 4) involving the right frontal lobe. Extended pathological examination was conducted in the Clinical Pathological Diagnosis & Research Center, Affiliated Hospital of the Youjiang Medical University for Nationalities. Molecular testing was performed using next-generation sequencing, which revealed mutations in *TP53 MSH2*, *MSH6, etc.* (**Table 1**) and microsatellite instability-high (MSI-H). Germline status was inferred based on variant pathogenicity classifications (ClinVar), Variant Allele Frequency (VAF) analysis, and clinical phenotype, in accordance with ESMO recommendations for tumor-only sequencing We then performed immunohistochemical staining of mismatch repair protein and found that MSH2 and MSH6 were missing (**Figures 3C–G**). Microsatellite instability status was determined using the MSI score, the results of 79 specific microsatellite loci, with instability at ≥30% defining MSI-H status (**Table 2**). Patient’s family history met Amsterdam II criteria: his father was diagnosed with colorectal cancer at age 36 and later developed gallbladder cancer, while a paternal cousin had early-onset colorectal cancer (age 42) (**Figure 4**). Although LS screening was not performed on the father’s tumor due to specimen unavailability, this clustering of malignancies strongly supported a hereditary predisposition. Postoperatively, the patient’s mental status improved from fair to near normal, and abnormal signals were not found in the MRI pictures (**Figure 5**). Currently, the patient is receiving concurrent treatment involving surgery intervention and intensity-modulated radiation therapy (IMRT) using 6MV-X radiation at a dose of DT 60 Gy. This combined modality treatment is being administered alongside temozolomide monotherapy for synchronized chemotherapy. Key hematological and biochemical parameters are summarized in **Table 3**. Follow-up evaluations will be conducted regularly to monitor the patient’s progress.

**Table 1:** Genomic test results by next generation sequencing.

Detected gene	Alterations	Alteration type	VAF or CN
TP53	NM_000546.6:c.1024C>T,p.R324^*^	Nonsense mutation	43.79%
TP53	NM_000546.6:c.743G>A,p.R248Q	Missense mutation	41.07%
BARD1	NM_000465.4:c.513del,p.D172Mfs^*^40	Frameshift deletion	20.98%
ATR	NM_001184.4:c.2320dup4,p.I774Nfs^*^3	Frameshift insertion	8.85%
MSH2	NM_000251.3:c.942 + 3A>T	Splice site mutation	46.03%
MSH6	NM_000179.3:c.3261dup,p.F1088Lfs^*^5	Frameshift insertion	14.07%
PDGFRA	amplification	Copy number gain	13.82

VAF, variant allele frequency; CN, copy number; NGS, next-generation sequencing;”*” indicates a stop codon.

**Table 2:** Microsatellite instability testing results.

Test items	MSI locus	MSS locus	Non-compliant locus	Score
MSI	MSI-30, MSI-72, MSI-27, MSI-73, MSI-61-MONO27, MSI-160, MSI-150, MSI-148, MSI-158, MSI-39, MSI-38, MSI-145, MSI-155, MSI-41, MSI-43, MSI-171, MSI-45, MSI-176, MSI-46, MSI-49, MSI-182, MSI-186, MSI-47, MSI-53, MSI-54, MSI-03-CAT25, MSI-197, MSI-58, MSI-77, MSI-78, MSI-91, MSI-90, MSI-101, MSI-63-NR22, MSI-105, MSI-06, MSI-106, MSI-108, MSI-09, MSI-111, MSI-115, MSI-17, MSI-15, MSI-134, MSI-135, MSI-129, MSI-131, MSI-170	MSI-26, MSI-28, MSI-64-NR24, MSI-157, MSI-142, MSI-165, MSI-140, MSI-48, MSI-184, MSI-52, MSI-194, MSI-55, MSI-193, MSI-198, MSI-200, MSI-75, MSI-85, MSI-07, MSI-109, MSI-62-NR21, MSI-110, MSI-11,MSI-114, MSI-14, MSI-128, MSI-124, MSI-119, MSI-20, MSI-208, MSI-60	MSI-02-BAT-26	48/78 = 0.6154>0.3

This method assesses microsatellite instability based on results from 79 specific microsatellite loci using a score calculated as: MSI Score = Number of unstable microsatellite loci/Number of effective loci microsatellite instability: Valid loci ≥ 20; MSI Score < 0.3 indicates microsatellite stability; MSI Score ≥ 0.3 indicates microsatellite instability.

**Table 3:** Longitudinal hematological and biochemical parameters at baseline and following concurrent chemoradiation.

Test items	Baseline (Pre-chemoradiation)	Post-chemoradiation	Unit	Reference range
White Blood Cells (WBC)	5.8	5.1	×10^9^/L	3.5-9.5
Neutrophils (NEU)	3.18	3.37	×10^9^/L	1.8-6.3
Lymphocyte (LYM)	1.77	0.86	×10^9^/L	1.1-3.2
Monocyte (MON)	0.54	0.51	×10^9^/L	0.1-0.6
Eosinophils (EOS)	0.25	0.36	×10^9^/L	0.02-0.52
Basophils (BAS)	0.07	0.03	×10^9^/L	0-0.06
Red blood cells (RBC)	5.15	6.44	×10^12^/L	4.3-5.8
Hemoglobin (HGB)	104	126	g/L	130-175
Hematocrit (HCT)	37.00	42.40	%	40-50
Mean Corpuscular Volume (MCV)	71.8	65.8	fL	82.0-100.0
Platelets (PLT)	271	257	×10^9^/L	125-350
Alanine aminotransferase (ALT)	16	28	U/mL	9-50
Aspartate aminotransferase (AST)	14	18	U/mL	15-40
Neuron-specific enolase (NSE)	12.00	12.68	ng/mL	0-20
Alpha-fetoprotein (AFP)	2.82	2.07	ng/mL	0-10
Carcinoembryonic Antigen (CEA)	1.21	1.01	ng/mL	0-5
Carbohydrate Antigen 19-9 (CA199)	11.95	<2.00	ng/mL	0-28

## 3 Discussion

In this case report, we present a case of glioblastoma occurring in a Chinese male patient with Lynch syndrome, characterized by pathogenic mutations in both the *MSH2* and *MSH6* genes. Our focus will be on investigating the manifestation of LS in brain tumors, specifically exploring its association with MMR and MSI. Additionally, we will discuss the therapeutic approaches undertaken for this patient.

Lynch syndrome (hereditary nonpolyposis colorectal cancer, HNPCC) is one of the most common hereditary cancer syndromes and is primarily associated with a high prevalence of colorectal and endometrial cancers. However, in addition to these classic tumor types, patients with Lynch syndrome may also have other extraintestinal tumors, including gastric, ovarian, and urinary tract cancers. The incidence of central nervous system tumors in patients with Lynch syndrome, although low, has been reported, particularly gliomas (glioma). Among these rare CNS tumors, GBM, on the other hand, exhibits a higher degree of malignancy as well as a poor prognosis.

Tumors in patients with Lynch syndrome are often associated with mutations in MMR genes, which are responsible for repairing errors in the DNA replication process. In turn, loss of function of MMR genes leads to microsatellite instability, a phenomenon that is common in many Lynch syndrome-associated tumors. Although *MSH2* mutations are one of the most common genetic alterations in LS, accounting for 35% of colorectal cancers according to MøllerP, SeppäläT et al. (11), coexisting MSH6 mutations are less common (12). The co-occurrence of *MSH2* and *MSH6* variants likely exerts a synergistic effect on genome-wide instability, amplifying both the overall cancer risk and the predisposition to aggressive phenotypes such as GBM. Both *MSH2* and *MSH6* genes encode mismatch repair proteins that play a crucial role in maintaining genome stability. *MSH2* is one of the most commonly mutated genes in Lynch syndrome and is responsible for encoding a protein that interacts with *MSH6* and *MSH3* to form the MutSα complex. This complex plays a crucial role in recognizing base mismatches and small insertion-deletion loops, thereby facilitating the repair of replication errors in DNA (13). If the mismatches are not repaired, which leads to MSI. Studies has shown that MSI-H is associated with an increased risk of developing multiple variant combinations (14). In our case, we observed similar findings. Thus, mutations in *MSH2* and *MSH6* result in defective DNA mismatch repair function, thereby increasing the risk of tumorigenesis.

**Figure 6:**
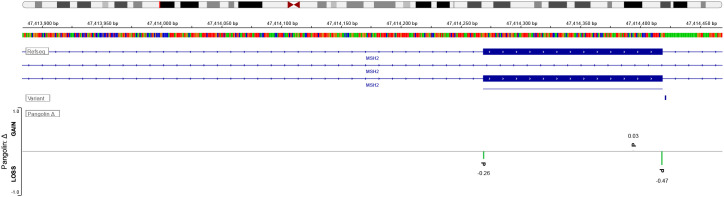
*In silico* splicing analysis of the *MSH2* variant using Pangolin. The deep-learning framework Pangolin was utilized to predict the impact of the identified germline variant (NM_000251.3:c.942 + 3A>T) on RNA splicing. The upper tracks display the RefSeq transcript isoforms for MSH2. The “PangolinΔ” track visualizes the predicted change in splice site usage probability (Delta score).

In this case, the mutation in *MSH2* was located in the intron 5 of the gene. Although intronic mutations typically do not directly affect protein coding, RNA analyses have shown that such variants can disrupt the RNA splicing process. This disruption leads to aberrant mRNA transcripts and, consequently, to functionally inactive proteins (15, 16). The location of intronic mutations in the *MSH2* gene is critical for Lynch syndrome, as these mutations can be pathogenic by affecting the mRNA splice site. Such mutations often lead to exon skipping or aberrant splicing, resulting in impaired mismatch repair proteins. Ultimately, this disruption leads to a loss of mismatch repair function. For this splicing variant, we performed multi-algorithm computational simulations and clinical functional validation. Using Pangolin, we identified that the NM_000251.3:c.942 + 3A>T variant induces splicing loss at the -3 bp position with a score of 0.47 (**Figure 6**). This position precisely corresponds to a classic splicing donor site at the exon-intron junction, indicating a significantly increased probability of disrupting the natural donor site. Clinically validated, this specific variant is a well-documented pathogenic mutation in Lynch syndrome (Variation ID: 36580). It has been classified as pathogenic by the expert panel InSiGHT. And the NM_000179.3:c.3261dup variant in this case is also considered pathogenic (Variation ID: 89364). Its location on exon 5 leads to a reading shift, which introduces a premature translation termination codon, resulting in a missing or interrupted protein product. Since the protein complexes of *MSH2* and *MSH6* work closely collaborate in DNA mismatch repair, the presence of both an intronic mutation in *MSH2* and a functional defect in MSH6 in this case further compromises the mismatch repair (MMR) system. This dual mutation significantly increases the risk of tumorigenesis, especially in rare CNS tumors such as glioblastoma, which can occur in the context of Lynch syndrome. The molecular characterization of this case not only confirms the diagnosis of LS but also guides therapeutic decision-making. Specifically, the identified dMMR and MSI-H status provide a rationale for exploring immune checkpoint inhibitors as an alternative to conventional alkylating chemotherapy.

In addition to MMR defects, somatic sequencing revealed a missense mutation in the *TP53*. This variant is located in exon 10 of the gene, causing the codon for arginine at position 342 in the encoded protein sequence to be replaced by a stop codon. This region also contains the oligomerization domain, which is crucial for p53 tetramerization. Truncating mutations in this domain typically result in loss of DNA-binding affinity and transcriptional activity, thereby disrupting p53-mediated cell cycle arrest and apoptosis mechanisms (17). In the context of Lynch syndrome, *TP53* mutations are not merely bystander events but frequently act as key co-drivers. Recent molecular characterization of MMR-deficient glioblastoma has identified a distinct high-risk subtype (G3/MMR), characterized by bilateral *TP53* inactivation and a histological pattern of multinucleated giant cells (18). This suggests a synergistic mechanism: *TP53* dysfunction enables survival of hypermutated cells with MMR deficiency, ultimately driving malignant.

At the therapeutic level, the molecular characteristics of LS-associated brain tumors offer potential breakthroughs for personalized treatment. Traditionally, standard therapeutic regimens for GBM (e.g., the STUPP regimen) have relied on the alkylating effect of temozolomide (TMZ) (2), but its efficacy is limited by the methylation status of the MGMT promoter and the functional integrity of the MMR. The absence of MGMT promoter methylation combined with dMMR in this case implies potential resistance to TMZ, consistent with reported mechanisms of alkylating agent resistance in MMR-deficient tumors (19). In recent years, immune checkpoint inhibitors (ICIs) have shown efficacy in dMMR tumors (20–22). While Tumor Mutational Burden (TMB) could not be directly quantified due to the limited genomic coverage of our targeted panel, the confirmed MSI-H status serves as a well-validated surrogate marker for a hypermutated tumor phenotype. In this context, dMMR drives widespread somatic mutations, particularly in repetitive genomic regions, resulting in elevated neoantigen production that enhances tumor immunogenicity. Consequently, despite the absence of a quantitative TMB value, this molecular profile strongly predicts favorable response to immune checkpoint inhibition with PD-1/PD-L1 inhibitors, consistent with established biomarker-guided treatment paradigms for MSI-H malignancies.

Although immunotherapy was not administered in this specific case due to clinical equipoise and guideline limitations at the time of treatment, our molecular characterization provides compelling evidence supporting its potential efficacy. The ‘gap’ in treating GBM lies in identifying immunogenic tumors. Our patient exhibited the hallmark MSI-High and dMMR phenotype. Previous pivotal trials, such as Keynote-158, have established that solid tumors with these specific biomarkers exhibit a pooled objective response rate (ORR) of approximately 34% to Pembrolizumab, regardless of histology (23). Furthermore, recent retrospective cohorts of Lynch-associated GBM have reported durable responses to immune checkpoint blockade, contrasting sharply with the failure of these drugs in unselected, sporadic GBM (18). The identification of this actionable signature in a 38-year-old patient underscores that the barrier to effective treatment is often diagnostic rather than therapeutic. The gap in knowledge is not if the drugs work (the mechanism is sound), but how to systematically identify the candidates. Our case highlights that limiting molecular investigation to standard markers (IDH/MGMT) fails to capture this distinct, treatable entity. It further underscores the importance of screening GBM patients for dMMR status to enable earlier transition to immune checkpoint inhibitor therapy.

Several limitations should be considered. First, the sample relied solely on tumor sequencing without paired germline confirmation. Germline status was inferred based on ClinVar, VAF analysis, clinical phenotype, and following ESMO recommendations, supplemented by immunohistochemistry. Second, the short postoperative follow-up period limits the assessment of long-term survival outcomes. Third, the lack of sequencing data restricts the validation of the presumed familial LS transmission. Cascade testing was strongly recommended for all first-degree relatives to assess their carrier status and initiate appropriate surveillance. However, at the time of this report, family members have declined sequencing due to financial constraints. Additionally, we plan to maintain long-term observation of this patient to monitor disease progression and treatment response.

In summary, this case highlights the need for genetic screening in the management of neurological tumors. Although brain tumors tend to be sporadic, systematic screening for MMR defects and LS-associated mutations should become a routine process in patients with a history of multiple primary tumors or familial cancer clustering, especially in patients with IDH wild-type GBM. Recent studies recommend mandatory assessment of MMR status in patients under 50 years of age with IDH wild-type GBM to rule out potential inherited syndromes. Neurosurgeons and oncologists must remain vigilant regarding hereditary syndromes, especially when encountering atypical or young-onset brain tumor cases. Interdisciplinary collaboration, such as involvement of genetic counseling teams, can significantly enhance diagnostic accuracy and treatment planning. Our findings bridge a knowledge gap by mechanistically linking co-mutations in *MSH2*/*MSH6* to gliomagenesis in LS patients. It supports the integration of routine MMR screening into standard treatment protocols to ensure future patients with this characteristic can access life-extending immunotherapy.

## 4 Conclusion

This case elucidates the pathogenic interplay between LS and GBM through concurrent germline *MSH2* and somatic *MSH6* mutations, which synergistically disrupt DNA MMR function, induce MSI-H, and drive gliomagenesis. The absence of MGMT promoter methylation and the presence MMR deficiency predict resistance to TMZ, while MSI-H status underscores the potential efficacy of immune checkpoint inhibitors as an alternative therapeutic strategy. Importantly, systematic genetic screening for MMR defects in young-onset or familial glioblastoma patients not only refines diagnostic accuracy but also facilitates personalized treatment and familial cancer surveillance. This emphasizes the clinical imperative of integrating molecular profiling into neuro-oncological practice.

## Data Availability

The original contributions presented in the study are included in the article/supplementary material. Further inquiries can be directed to the corresponding author.

## References

[1] Louis DN Perry A Wesseling P Brat DJ Cree IA Figarella-Branger D . The 2021 WHO classification of tumors of the central nervous system: a summary. Neuro Oncol. (2021) 23:1231–51. doi: 10.1093/neuonc/noab106, PMID: 34185076 PMC8328013

[2] Stupp R Mason WP van den Bent MJ Weller M Fisher B Taphoorn MJ . Radiotherapy plus concomitant and adjuvant temozolomide for glioblastoma. N Engl J Med. (2005) 352:987–96. doi: 10.1056/NEJMoa043330, PMID: 15758009

[3] Rong L Li N Zhang Z . Emerging therapies for glioblastoma: current state and future directions. J Exp Clin Cancer Res. (2022) 41:142. doi: 10.1186/s13046-022-02349-7, PMID: 35428347 PMC9013078

[4] Samadder NJ Smith KR Wong J Thomas A Hanson H Boucher K . Cancer risk in families fulfilling the amsterdam criteria for lynch syndrome. JAMA Oncol. (2017) 3:1697–701. doi: 10.1001/jamaoncol.2017.0769, PMID: 28772302 PMC5824265

[5] Win AK Lindor NM Young JP Macrae FA Young GP Williamson E . Risks of primary extracolonic cancers following colorectal cancer in lynch syndrome. J Natl Cancer Inst. (2012) 104:1363–72. doi: 10.1093/jnci/djs351, PMID: 22933731 PMC3529597

[6] Peltomäki P Nyström M Mecklin JP Seppälä TT . Lynch syndrome genetics and clinical implications. Gastroenterology. (2023) 164:783–99. doi: 10.1053/j.gastro.2022.08.058, PMID: 36706841

[7] Fishel R . Mismatch repair. J Biol Chem. (2015) 290:26395–403. doi: 10.1074/jbc.R115.660142, PMID: 26354434 PMC4646297

[8] Hause RJ Pritchard CC Shendure J Salipante SJ . Classification and characterization of microsatellite instability across 18 cancer types. Nat Med. (2016) 22:1342–50. doi: 10.1038/nm.4191, PMID: 27694933

[9] Alnahhas I Rayi A Ong S Giglio P Puduvalli V . Management of gliomas in patients with lynch syndrome. Neuro Oncol. (2021) 23:167–68. doi: 10.1093/neuonc/noaa227, PMID: 33059358 PMC7850082

[10] Therkildsen C Ladelund S Rambech E Persson A Petersen A Nilbert M . Glioblastomas, astrocytomas and oligodendrogliomas linked to lynch syndrome. Eur J Neurol. (2015) 22:717–24. doi: 10.1111/ene.12647, PMID: 25648859

[11] Møller P Seppälä T Bernstein I Holinski-Feder E Sala P Evans DG . Cancer incidence and survival in lynch syndrome patients receiving colonoscopic and gynaecological surveillance: first report from the prospective lynch syndrome database. Gut. (2017) 66:464–72. doi: 10.1136/gutjnl-2015-309675, PMID: 26657901 PMC5534760

[12] Hampel H Frankel WL Martin E Arnold M Khanduja K Kuebler P . Feasibility of screening for lynch syndrome among patients with colorectal cancer. J Clin Oncol. (2008) 26:5783–88. doi: 10.1200/JCO.2008.17.5950, PMID: 18809606 PMC2645108

[13] Jiricny J . The multifaceted mismatch-repair system. Nat Rev Mol Cell Biol. (2006) 7:335–46. doi: 10.1038/nrm1907, PMID: 16612326

[14] Arnold S Buchanan DD Barker M Jaskowski L Walsh MD Birney G . Classifying MLH1 and MSH2 variants using bioinformatic prediction, splicing assays, segregation, and tumor characteristics. Hum Mutat. (2009) 30:757–70. doi: 10.1002/humu.20936, PMID: 19267393 PMC2707453

[15] Liu B Parsons RE Hamilton SR Petersen GM Lynch HT Watson P . HMSH2 mutations in hereditary nonpolyposis colorectal cancer kindreds. Cancer Res. (1994) 54:4590–94. 8062247

[16] Skeldon SC Semotiuk K Aronson M Holter S Gallinger S Pollett A . Patients with lynch syndrome mismatch repair gene mutations are at higher risk for not only upper tract urothelial cancer but also bladder cancer. Eur Urol. (2013) 63:379–85. doi: 10.1016/j.eururo.2012.07.047, PMID: 22883484

[17] Yamamoto S Iwakuma T . Regulators of oncogenic mutant TP53 gain of function. Cancers (Basel). (2018) 11:4. doi: 10.3390/cancers11010004, PMID: 30577483 PMC6356290

[18] Georgescu M . Adult glioblastoma with lynch syndrome-associated mismatch repair deficiency forms a distinct high-risk molecular subgroup. Free Neuropathol. (2024) 5:32. doi: 10.17879/freeneuropathology-2024-5892, PMID: 39835141 PMC11745196

[19] Iturrioz-Rodríguez N Sampron N Matheu A . Current advances in temozolomide encapsulation for the enhancement of glioblastoma treatment. Theranostics. (2023) 13:2734–56. doi: 10.7150/thno.82005, PMID: 37284445 PMC10240814

[20] Marabelle A Le DT Ascierto PA Di Giacomo AM De Jesus-Acosta A Delord JP . Efficacy of pembrolizumab in patients with noncolorectal high microsatellite instability/mismatch repair-deficient cancer: results from the phase II KEYNOTE-158 study. J Clin Oncol. (2020) 38:1–10. doi: 10.1200/JCO.19.02105, PMID: 31682550 PMC8184060

[21] Le DT Uram JN Wang H Bartlett BR Kemberling H Eyring AD . PD-1 blockade in tumors with mismatch-repair deficiency. N Engl J Med. (2015) 372:2509–20. doi: 10.1056/NEJMoa1500596, PMID: 26028255 PMC4481136

[22] Hadad S Gupta R Oberheim BN Taylor JW Villanueva-Meyer JE Young JS . *De novo* replication repair deficient glioblastoma, IDH-wildtype” is a distinct glioblastoma subtype in adults that may benefit from immune checkpoint blockade. Acta Neuropathol. (2023) 147:3. doi: 10.1007/s00401-023-02654-1, PMID: 38079020 PMC10713691

[23] O’Malley DM Bariani GM Cassier PA Marabelle A Hansen AR De Jesus Acosta A . Health-related quality of life with pembrolizumab monotherapy in patients with previously treated advanced microsatellite instability high/mismatch repair deficient endometrial cancer in the KEYNOTE-158 study. Gynecol Oncol. (2022) 166:245–53. doi: 10.1016/j.ygyno.2022.06.005, PMID: 35835611

